# Prospects of Coffee Leaf against SARS-CoV-2 Infection

**DOI:** 10.7150/ijbs.76058

**Published:** 2022-07-11

**Authors:** Chen-Shiou Wu, Hsiu-Mei Chiang, Yeh Chen, Chung-Yu Chen, Hsiao-Fan Chen, Wen-Chi Su, Wei-Jan Wang, Yu-Chi Chou, Wei-Chao Chang, Shao-Chun Wang, Mien-Chie Hung

**Affiliations:** 1 Graduate Institute of Biomedical Sciences, China Medical University, Taichung 406040, Taiwan.; 2 Department of Cosmeceutics, China Medical University, Taichung 406040, Taiwan.; 3 Institute of New Drug Development, China Medical University, Taichung 406040, Taiwan.; 4 Research Center for Cancer Biology, China Medical University, Taichung 406040, Taiwan.; 5 International Master's Program of Biomedical Sciences, China Medical University, Taichung 406040, Taiwan.; 6 Department of Medical Research, China Medical University Hospital, Taichung 404332, Taiwan.; 7 Department of Biological Science and Technology, China Medical University, Taichung 406040, Taiwan.; 8 Biomedical Translation Research Center (BioTReC), Academia Sinica, Taipei 115024, Taiwan.; 9 Center for Molecular Medicine, China Medical University Hospital, China Medical University, Taichung 404332, Taiwan.; 10 Department of Biotechnology, Asia University, Taichung, 41354 Taiwan.

**Keywords:** Coffee leaf, SARS-CoV-2, Spike, ACE2, skin

## Abstract

In the current climate, many countries are in dire need of effective preventive methods to curb the Severe Acute Respiratory Syndrome Coronavirus Type 2 (SARS-CoV-2) pandemic. The purpose of this research is to screen and explore natural plant extracts that have the potential to against SARS-CoV-2 and provide alternative options for SARS-CoV-2 prevention and hand sanitizer or spray-like disinfectants. We first used Spike-ACE2 ELISA and TMPRSS2 fluorescence resonance energy transfer (FRET) assays to screen extracts from agricultural by-products from Taiwan with the potential to impede SARS-CoV-2 infection. Next, the SARS-CoV-2 pseudo-particles (Vpp) infection assay was tested to validate the effectiveness. We identified an extract from coffee leaf (*Coffea Arabica*), a natural plant that effectively inhibited wild-type SARS-CoV-2, and five Variants of Concern (Alpha, Beta, Gamma, Delta, and Omicron strain) from entering host cells. In an attempt to apply coffee leaf extract for hand sanitizer or spray-like disinfectants, we designed a skin-like gelatin membrane experiment. We showed that the high concentration of coffee leaf extract on the skin surface could block SARS-CoV-2 into cells more potently than 75% Ethanol, a standard disinfectant to inactivate SARS-CoV-2. Finally, LC-HRMS analysis was used to identify compounds such as caffeine, chlorogenic acid (CGA), quinic acid, and mangiferin that are associated with an anti-SARS-CoV-2 activity. Our results demonstrated that coffee leaf extract, an agricultural by-product effectively inhibits SARS-CoV-2 Vpp infection through an ACE2-dependent mechanism and may be utilized to develop products against SARS-CoV-2 infection.

## Introduction

Coronavirus is a group of viruses that can cause respiratory infections in humans 1. At the end of 2019, a new coronavirus species named SARS-CoV-2 caused the Coronavirus Disease 2019 (COVID-19), resulting in deaths or long-term conditions from the infection (more than pneumonia, COVID-19 exhibits various life-threatening symptoms). Countries have reported large-scale infection clusters, added up with the high transmission efficiency and a large amount of international travel; therefore, SARS-CoV-2 presents a huge threat to global public health 2, 3.

SARS-CoV-2 is an enveloped single-stranded RNA (ssRNA) virus including four crucial proteins, namely, spike (S), membrane (M), nucleocapsid (N), and envelope (E) protein 4. It was shown that the Spike binds to the angiotensin-converting enzyme 2 (ACE2) on the surface of the host cell and is further triggered by the transmembrane protease serine 2 (TMPRSS2) 5. Despite masks and other safety measures, millions of people were infected with COVID-19 leading to unprecedented mortality. Researchers have designed and developed new classes of vaccines based on the structure of SARS-CoV-2 to curb this global pandemic 6. However, due to underlining health conditions and other unknown factors, breakthrough cases occur in the fully vaccinated population 7. In addition, the mutations of SARS-CoV-2 enhance viral transmission and reduce the efficacy of current vaccines 8, 9. The WHO has recognized five SARS-CoV-2 variants of concern (VOC) at https://www.who.int/en/activities/tracking-SARS-CoV-2-variants/. The Alpha strain (B.1.1.7) was first discovered in the UK, the Beta strain (B.1.351) was detected in South Africa, and the Gamma strain (P.1) was reported in Brazil that they had been found to spread faster than other mutants 10-12. The Alpha strain has exhibited a stronger transmission rate and infection rate than the other two 13 till the Delta strain (B.1.617.2) 14 replaces the alpha strain becoming the dominant strain. Researchers discovered the Omicron strain (B.1.1.529), which appeared in November 2021 from South Africa. It has more than thirty changes to the spike protein, and the changes have been found in variants such as Delta and Alpha. These changes are related to the increase in transmissibility, higher viral binding affinity, and antibody escape 15, 16. Serious threat to current COVID-19 vaccines and therapies, potentially pressing humans to coexist with COVID-19, and the standards of disinfection and social distancing have gradually become an important new area for epidemic prevention 17, 18. Therefore, other preventive approaches to reduce the transmission of all SARS-CoV-2 variants 19 are urgently needed in addition to vaccination.

Various studies have shown the anti- SARS-CoV-2 activity of plant extracts, such as *Scutellaria barbata* D. Don 20, *Allium sativum*, *Camellia sinensis* 21, 22, and* Punica granatum* 23, 24. Some natural plants from the economic crops, such as barks, leaves, roots, and others, have the potential to become high-value compounds. Coffee leaf from coffee production is a great example 25. For a long time, in India, Jamaica, and Java, the coffee leaf has been used to improve health as ethnomedicine, animal feed, lactobacillus proliferators, and organic fungicides 26. The component of the coffee leaf has many distinct phytochemicals with a variety of potential biological activities, such as amino acids, alkaloids (caffeine), flavonoids (anthocyanins), xanthonoids (mangiferin), tannins, phenolic acids (caffeic acid, chlorogenic acid), and catechins 27, 28. For example, mangiferin has exhibited anti-inflammatory, anti-diabetic, anti-hyperlipidemia, anti-oxidation, and neuroprotective effects; chlorogenic acid can inhibit oxidative stress, inflammation, aging, and cancer 29-32.

This report utilized relevant cell-free assays and pseudovirus infection assays to examine a series of natural plants from economic crops in Taiwan. We identified coffee leaf as a promising alternative source that interferes with SARS-CoV-2 in the prevention and phytotherapy of medicine.

## Materials and methods

### Extraction of natural plants

The leaves and stems of the plants were collected and dried at 50°C. Next, the dried plants were weighted at a ratio of 1:30, soaked in different proportions of ethanol or methanol, and homogenized by ultrasonic vibration for 1 hour. After the second extraction in the above steps, freeze crystals were dried and re-dissolved to 1 mg/ml with water to obtain natural plant extracts. All extracts were stored at -20°C. Details were described in the previous report 33, 34.

### Spike-ACE2 binding assay

The SARS-CoV-2 Neutralizing Antibody ELISA Kit (Spike-ACE2 Binding Assay Kit II, CoV-SACE2-1, RayBiotech Life, Peachtree Corners, GA, USA) is an enzyme-linked immunosorbent assay (ELISA), was used to screen potential natural plant extracts neutralized SARS-CoV-2 Spike binding with host cell ACE2 protein following the protocol provided by the manufacturer.

### Inhibition of TMPRSS2 activity by fluorescence resonance energy transfer (FRET) assay

The experiment that determined the activity of different concentrations of the grape stem (*Vitis vinifera*), gardenia leaf (*Gardenia jasminoides*), and coffee leaf (*Coffea Arabica*) extracts to inhibit TMPRSS2 was in reference 35. With or without extracts, the 25 μM TMPRSS2 was pre-incubated for a half-hour at room temperature. Next, 20 μM fluorescent protein substrate was added and measured by Synergy™ H1 hybrid multi-mode microplate reader (BioTek Instruments, Inc., Winooski, VT, USA). The reaction was continuously monitored at the emission of 536 nm.

### SARS-CoV-2 pseudo-particles (Vpp) infection assay

Wild-type and five VOC (Alpha, Beta, Gamma, Delta and Omicron strain) SARS-CoV-2 pseudo-particles were purchased from RNAi core, Academia Sinica, Taiwan. HEK293T cells (the human embryonic kidney cell line) overexpressing ACE2 (293T-ACE2) were maintained in Dulbecco's MEM containing 10% fetal bovine serum and 1% penicillin/streptomycin. And, Calu-3 cells (human lung cancer cell line) were cultured in Dulbecco's MEM supplemented with 10% fetal bovine serum, 1× NEAA, and 1% penicillin/streptomycin. 10^4^ (293T-ACE2) and 5 × 10^3^ (Calu-3) cells were seeded into 96-well plates, and 4-100 μg/ml of the grape stem (*Vitis vinifera*), 4-100 μg/ml of gardenia leaf (*Gardenia jasminoides*), and 4-100 μg/ml of coffee leaf (*Coffea Arabica*) extracts, or H_2_O (control) in a medium were pretreated for 1 hour. Then, treated cells were infected with 50 μl of medium containing pseudo-viral particles (MOI = 0.2). After overnight incubation, cell viability was measured with a 10 μl Cell Counting Kit-8 (CCK-8) (Dojindo Laboratories, Japan) and incubated for 2 hours. Next, each sample was mixed with the same volume of Bright-Glo Luciferase Detection System (Promega, USA) for 10 minutes. The luciferase activity was detected through the GloMax Navigator System (Promega, USA). The relative light unit (RLU) normalized cell viability represents the relative infection efficiency. More details were described in references 20, 23.

### Skin-like gelatin membranes test

According to a report by Alarcón-Segovia *et al.* 36, 2.5 g gelatin from bovine skin (Sigma-Aldrich, USA) with glycerol and MilliQ water in a 12-well culture plate into a thickness of 1-2 mm were maturated 15 minutes as forming polymer. 5 μl of 100 μg/ml coffee leaf (*Coffea Arabica*) extract, 75% (weight/weight [w/w]) Ethanol, or H_2_O (control) were applied separately on skin-like gelatin membranes at room temperature from 15 minutes to 1 hour and added into 2 μl pseudo-viral particles (MOI=0.2) for 2 hours. Subsequently, they were added with 50 μl of medium for the Vpp infection assay.

### LC-HRMS analysis

To a previous report 20, the aliquots extracts were dissolved in methanol at 10 μg/ml and conducted on a Q-Exactive Plus orbitrap high-resolution mass spectrometer equipped with an Ultimate 3000 ultra-high performance liquid chromatographic (UHPLC) system (ThermoScientific, San Jose, CA, USA) and a Waters ACQUITY UPLC C18 column (2.1 × 100 mm, 1.7 um). The adopted gradient was set as follows: from 5% buffer B at 1 minute to 30% buffer B at 15 minutes, subsequently to 80% buffer B at 16 minutes, and maintained at 80% buffer B for 5 minutes, where buffer A was 0.2% formic acid/H2O, and buffer B was acetonitrile. The flow rate of 0.4 mL/min. The parallel reaction monitoring (PRM) mode of Q-Exactive Plus was utilized to characterize chemical components and quantified caffeine, chlorogenic acid (CGA), quinic acid, and mangiferin. The survey scan was conducted in the m/z 150-2000, with an electrospray voltage of 4.0 kV and 3.5 kV for positive and negative ionization modes, respectively. The standard pure compounds, caffeine, chlorogenic acid, and quinic acid, were purchased from Sigma-Aldrich, USA. The mangiferin was acquired from ChromaDex, California, USA.

### SARS-CoV-2 Spike S1:ACE2 time resolved-fluorescence resonance energy transfer (TR-FRET) assay

The inhibitory effect of the main compounds of the coffee leaf (*Coffea Arabica*) to inhibit the binding between SARS-CoV-2 Spike S1 and human ACE2 were measured by SARS-CoV-2 Spike S1-Biotin: ACE2 TR-FRET Assay Kit (#79949-1, San Diego, CA, USA). The procedure had followed the commercial protocol. The main test compounds, 100 μg/ml caffeine, 100 μg/ml CGA, 100 μg/ml quinic acid, and 100 μg/ml mangiferin, were incubated with Spike S1-Biotin, and ACE2-Eu, dye-labeled acceptor, respectively for 1 hour. Then the TR-FRET signal was measured in a microtiter-plate reader (BioTek Instruments, Inc., Winooski, VT, USA).

### Data analysis

All data in this study were presented as the mean ± SEM in triplicate independent experiments. Statistical analysis was applied by analysis of variance (ANOVA) and Student *T*-test. *,* P* < 0.05, **, *P* < 0.01 or ***,* P* < 0.001 is concerned statistically significant.

## Results

### Coffee leaf extract inhibits binding between Spike protein and ACE2 and enzymatic activity of TMPRSS2

**Figure 1:**
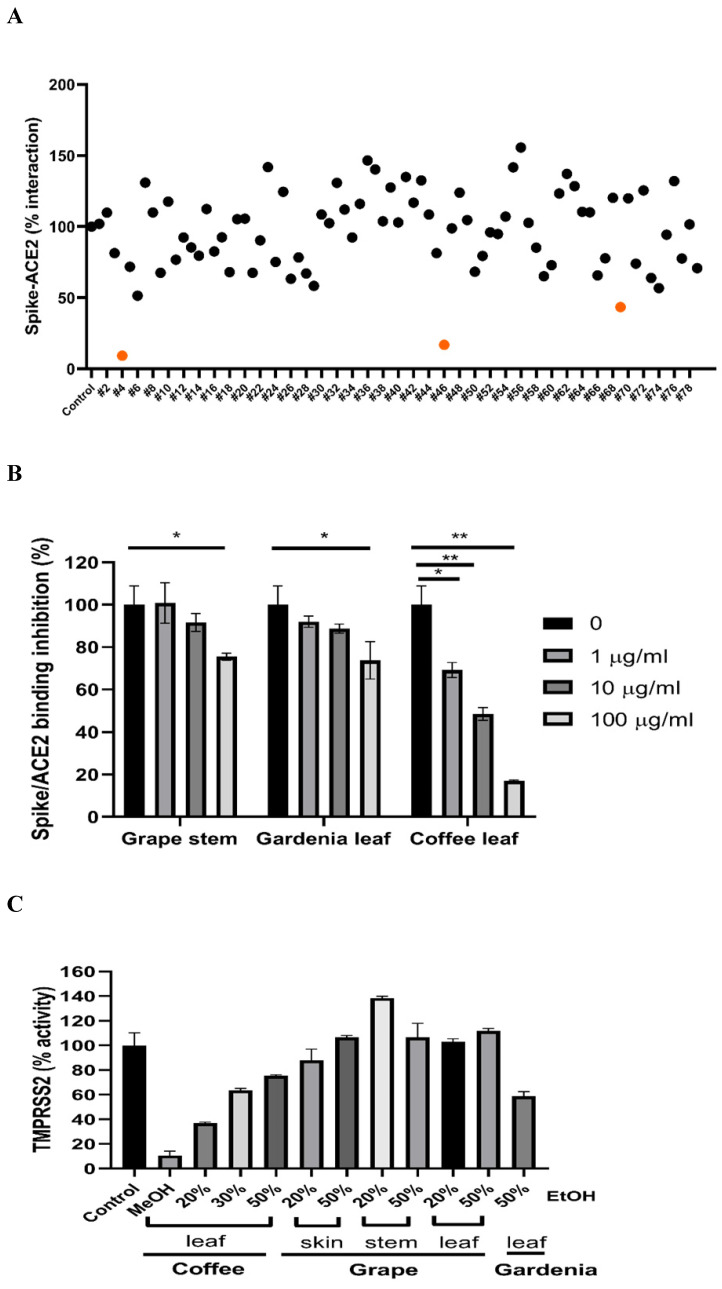
Identification of potential nature plants against both SARS-CoV-2 Spike-ACE2 interaction and enzymatic activity of TMPRSS2. (A)A commercial SARS-CoV-2 Neutralizing Antibody ELISA Kit (detecting Spike-ACE2 protein interaction) was used to test the effectiveness in different alcohol ratios or methanol-extracted natural plants. (B)The effectiveness of the grape stem (*Vitis vinifera*), gardenia leaf (*Gardenia jasminoides*), and coffee leaf (*Coffea Arabica*) extracts was confirmed by SARS-CoV-2 Neutralizing Antibody ELISA Kit. (C)The effect of the grape stem (*Vitis vinifera*), gardenia leaf (*Gardenia jasminoides*), and coffee leaf (*Coffea Arabica*) extracts on inhibiting TMPRSS2 activity was tested by FRET assay. Data were presented as mean ± SEM executed in triplicate. * P < 0.05, ** *P* < 0.01, *** P < 0.001.

To test if the extracts from natural plants may associate with anti-SARS-CoV-2 activity, first, we tested Spike-ACE2 interaction by cell-free ELISA assay. In this study, we extracted natural plants with 20-50% (w/w) ethanol (EtOH), water, or methanol (MeOH), respectively. The powder obtained by freeze-drying was dissolved in water to form a final concentration of 1 mg/ml. ELISA assay detecting Spike RBD binding to hACE2 37 was used to screen our current natural plant extracts **(Supplementary Table 1).** Most extracts screened at 100 μg/ml indicated no notable effect to disrupt the Spike-ACE2 binding. Grape stem (*Vitis vinifera*), gardenia leaf (*Gardenia jasminoides*) with 50% EtOH, and coffee leaf (*Coffea Arabica*) with MeOH had an obvious neutralization activity **(Figure 1A)**. Concentration-dependent assays of the grape stem, gardenia leaf, and coffee leaf extract further validated that extract of the coffee leaf (*Coffea Arabica*) exhibited the strongest neutralization activity at high concentration (up to 83% inhibition) **(Figure 1B)**. Next, we asked whether these natural plants may affect the activity of TMPRSS2 known to influence the entry of SARS-CoV-2 into cells. The in vitro FRET-based enzyme analysis of TMPRSS2 35 showed that treatment with coffee leaf (*Coffea Arabica*) with MeOH strongly inhibited the activity of TMPRSS2 **(Figure 1C)**. In contrast, the TMPRSS2 enzyme activity assay showed that grape stem (*Vitis vinifera*), and gardenia leaf (*Gardenia jasminoides*) with 50% EtOH had poor inhibition **(Figure 1C)**. The results indicated that coffee leaf extract in MeOH could inhibit both Spike-ACE2 interaction and enzymatic activity of TMPRSS2, the two critical activities for the SARS-C-V-2 viral entrance. However, grape stem (*Vitis vinifera*), and gardenia leaf (*Gardenia jasminoides*) with 50% EtOH could inhibit only Spike-ACE2 interaction.

### Coffee leaf extract has an inhibitory potential against SARS-CoV-2 infection

**Figure 2:**
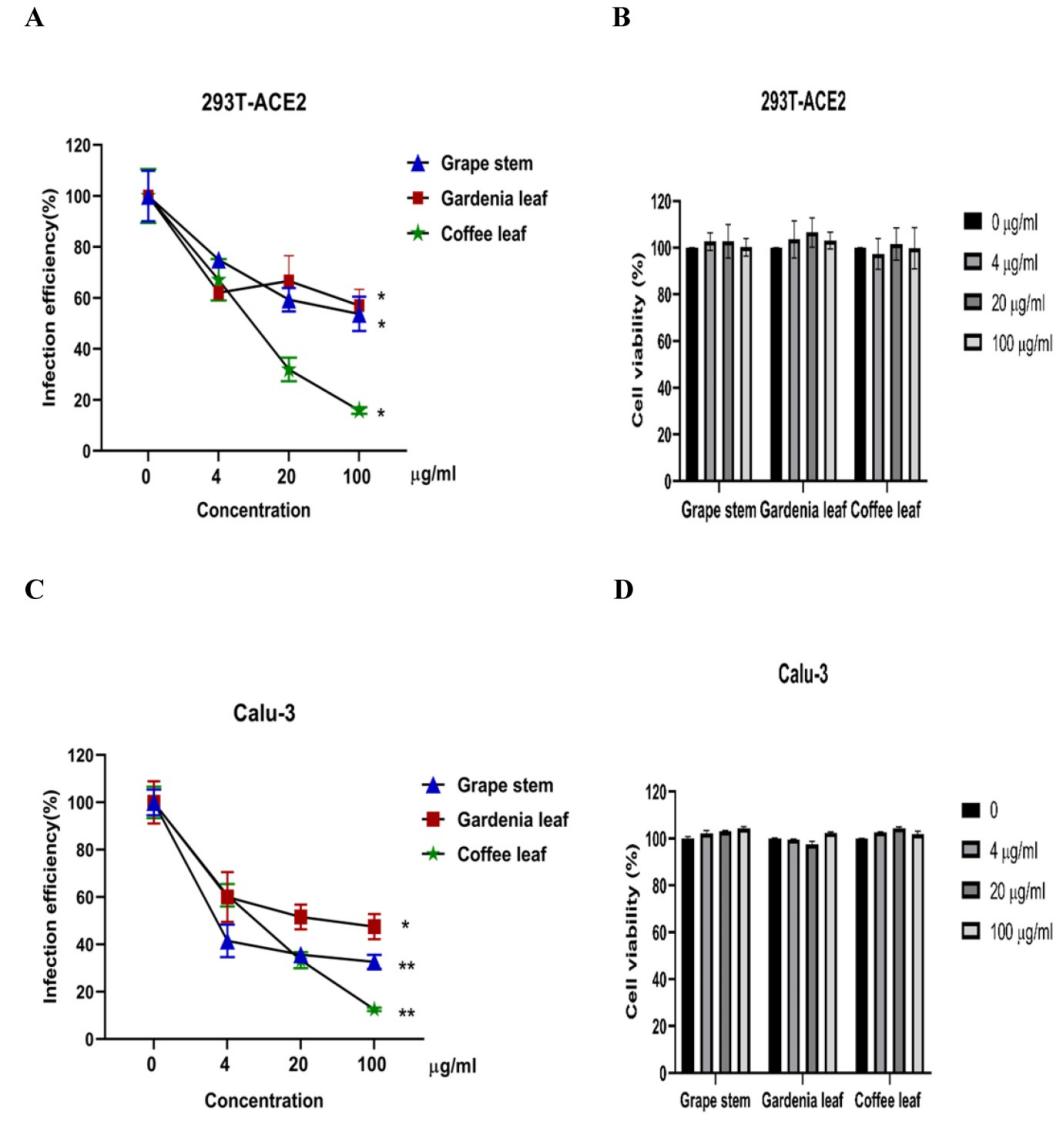
The inhibitory effect of the grape stem, gardenia leaf, and coffee leaf extracts on SARS-CoV-2 pseudo-particles (Vpp) infection. (A) The coffee leaf extract showed dose-dependent inhibition in wild-type SARS-CoV-2 Vpp infection on 293T-ACE2 cells. (B) 293T cells with ACE2 over-expression were treated with the indicated concentrations of the grape stem (Vitis vinifera), gardenia leaf (Gardenia jasminoides), and coffee leaf (Coffea Arabica) extracts for 24 hours measured by CCK-8 assay demonstrated no cytotoxicity. (C) The high concentration of three natural plant extracts significantly inhibited wild-type SARS-CoV-2 Vpp infection on Calu-3 cells. (D) Three natural plant extracts were treated on Calu-3 cells for 24 hours measured by CCK-8 assay demonstrated no cytotoxicity. Data were presented as mean ± SEM in triplicate. * P < 0.05, ** *P* < 0.01.

**Figure 3:**
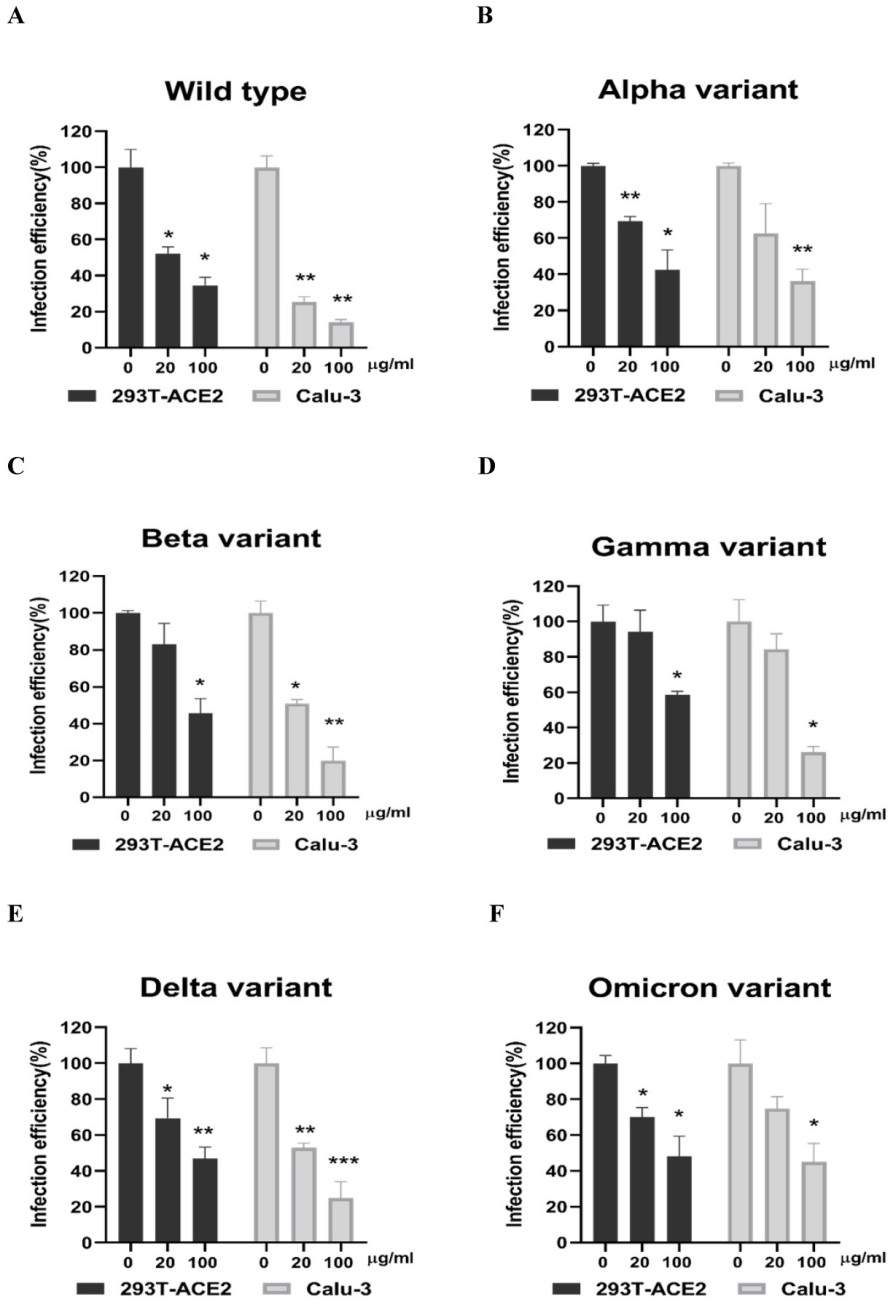
The coffee leaf extract had the potent capacity to restrain five VOC SARS-CoV-2 Vpp infections. (A-F) 293T-ACE2 cells and Calu-3 cells were treated with the indicated concentrations of coffee leaf (Coffea Arabica) extracts after 24 hours of infection and measured by luciferase activity. The high concentration of coffee leaf extract had a significant effect on inhibiting five VOC SARS-CoV-2 infections of 293T-ACE2 cells and Calu-3 cells. Statistical significance was measured by Student* t*-test, * P < 0.05, ** *P* < 0.01, *** P < 0.001.

Next, we evaluated whether grape stem, gardenia leaf, and coffee leaf extracts could inhibit the pseudoviral SARS-CoV-2 (Vpp) Spike mediated entry. We selected two human cell lines with the pseudovirus system to assess their susceptibility to SARS-CoV-2 Spike-mediated entry. We used 293T-ACE2 cells 38, 293T cells stably transfected with human ACE2 to facilitate SARS-CoV-2 entering into target cells and the human lung cancer cell line Calu-3 that is highly sensitive to SARS-CoV-2 Spike-mediated entry 5. The coffee leaf extract inhibited the viral entrance activity by Vpp assay was stronger than the grape stem and gardenia leaf extracts, likely due to coffee leaf extract possessing dual activities shown in **Figure 2A, C**. The cell viability results showed no significant cytotoxicity by CCK-8 assay after incubation for 24 hours **(Figure 2B, D)**, indicating the suppressed infection activity was not caused by cell viability. We further compared the inhibitory activity of coffee leaf extract on the five VOC (Alpha, Beta, Gamma, Delta, and Omicron strain) Vpp. The inhibitory activity of coffee leaf extract on the four variants is not as potent as that on the wild type; however, at 100 μg/ml, coffee leaf extract still had a significant inhibitory effect on the infection of five VOC Vpp **(Figure 3A-F)**. Yet grape stem and gardenia leaf were unable to effectively suppress the five VOC Vpp on 293T-ACE2 cells **(Supplementary Figure 1)**. Thus, the results suggest that coffee leaf extract can block the infection of wild-type, and its effect is still effective but less potent on the four variants of pseudo-viral SARS-CoV-2.

### Coffee leaf extract has the potential to develop into daily necessities through inhibiting SARS-CoV-2

**Figure 4:**
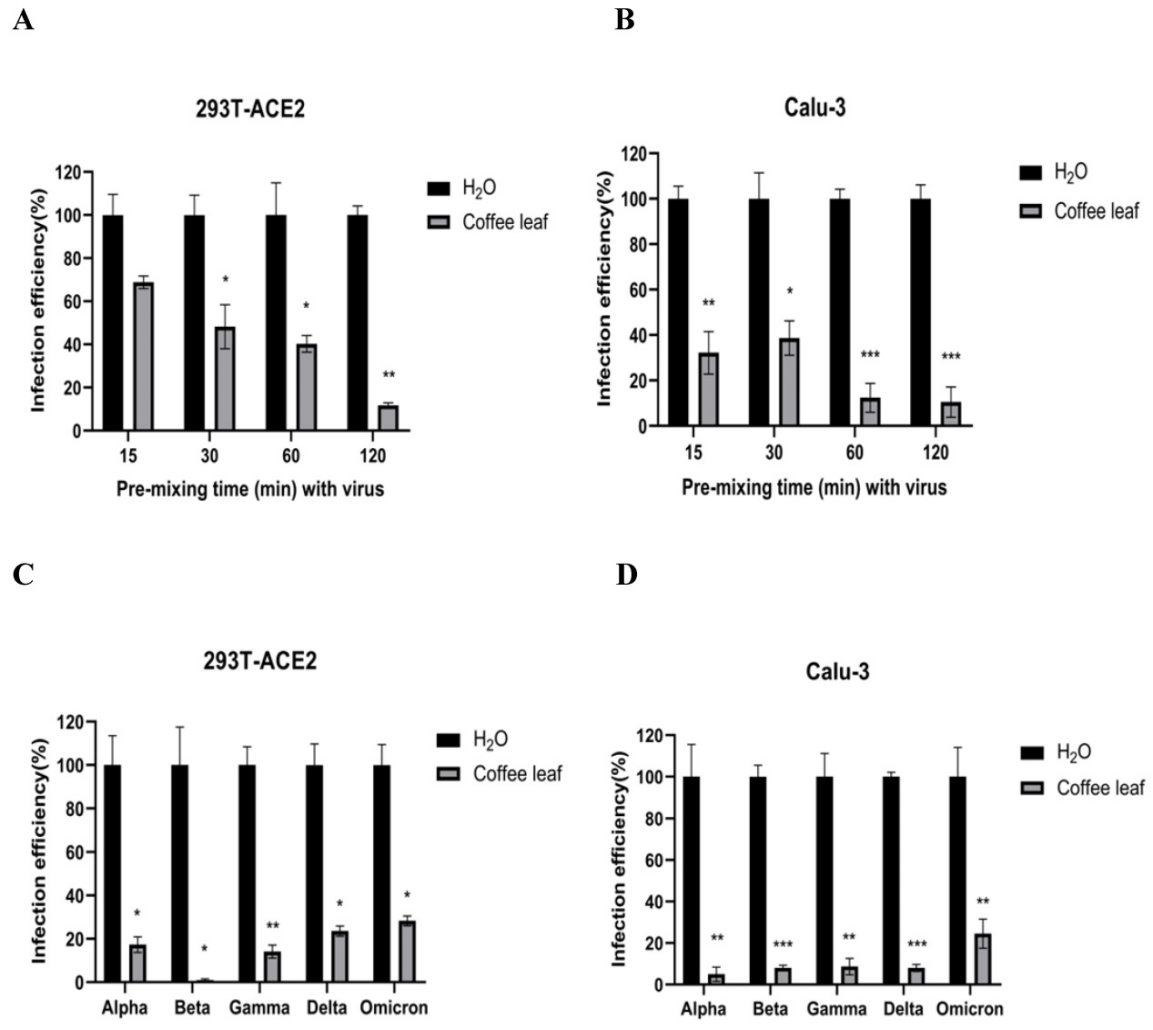
The effect of coffee leaf extract blocks wild-type and five VOC SARS-CoV-2 Vpp infection (virus pre-treatment). (A) 100 μg/ml coffee leaf extract could block the entry of wild-type SARS-CoV-2 Vpp (virus pre-treatment) in a time-dependent manner on 293T-ACE2 cells. (B) 100 μg/ml coffee leaf extract had the stronger effect of inhibiting the entry of wild-type SARS-CoV-2 Vpp (virus pre-treatment) in a time-dependent manner on Calu-3 cells. (C) 100 μg/ml coffee leaf extract can inhibit five VOC SARS-CoV-2 Vpp (virus pre-treatment) infections significantly on 293T-ACE2 cells. (D) 100 μg/ml coffee leaf extract can inhibit five VOC SARS-CoV-2 Vpp (virus pre-treatment) infections remarkably on Calu-3 cells. Data were presented as mean ± SEM in triplicate. * P < 0.05, ** *P* < 0.01, *** P < 0.001.

The cell viability results in **Figure 2B and 2D** prompted us to test if coffee leaf extract could be safely used, such as hand sanitizer or spray-like disinfectants, due to its ability to inhibit SARS-CoV-2 infection. First, we measured whether it has the activity of restraining Vpp infection in the state of extract synchronized mix with the virus and then put it into 293T-ACE2 cells and Calu-3 cells (virus pre-treatment assay). Experimental results showed that 100 μg/ml coffee leaf extract could effectively inhibit the entry of wild-type Vpp in a time-dependent manner (**Figure 4A, B**). Importantly, within 2 hours of incubation, the wild-type virus infectivity was reduced to less than 12%. In addition, it can also strongly restrain the five VOC Vpp within 2 hours of incubation (**Figure 4C, D**).

**Figure 5:**
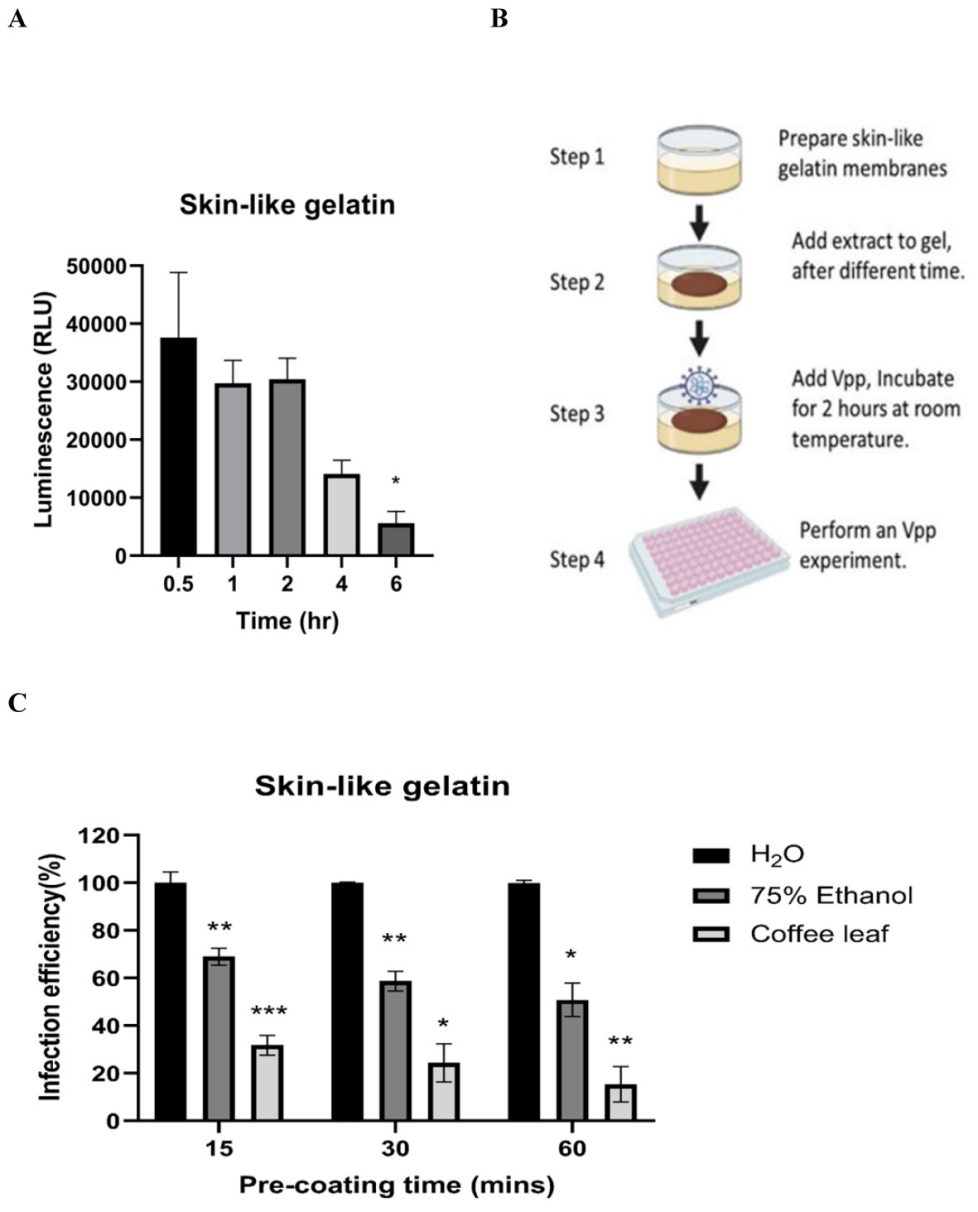
The coffee leaf extract inhibited SARS-COV-2 Vpp on the skin-like gelatin membranes. (A) The SARS-CoV-2 Vpp infection was tested for 0.5 to 6 hours. And, there was no significant difference in its infection within 2 hours of exposure, but it presented a remarkable reduce after 6 hours of exposure. (B) The models of skin-like gelatin membranes for testing. (C) Coffee leaf extract could inhibit the wild-type SARS-CoV-2 Vpp on the surface of the skin-like gelatin membrane within 60 minutes. And, its inhibiting effectiveness was better than 75% (w/w) Ethanol under a long exposure time. Data were presented as mean ± SEM in triplicate. * P < 0.05, ** *P* < 0.01, *** P < 0.001.

Next, we tested whether coffee leaf extract has the potential to be added for hand sanitizer or spray-like disinfectants to usefully avoid SARS-CoV-2 infection through skin contact. To this end, we first tested how long the Vpp can stay on the surface of skin-like gelatin membranes. As shown in **Figure 5A**, Vpp infection efficiency was gradually lost after 6 hrs; however, within 1-2 hrs, Vpp remained most of the infection activity. The results from **Figure 4 and 5A** allow us to design a procedure as shown in **Figure 5B** to test the efficacy of coffee leaf extract to block Vpp infectivity, where the 2 hr incubation time with Vpp at step 3 was required to stop the majority of activity based on the results from the **Figure 4**. The activity of 75% Ethanol, a standard disinfectant, was compared with that of the coffee leaf extract. 5 μl of 100 μg/ml coffee leaf (*Coffea Arabica*) extract, 75% (w/w) Ethanol, or H_2_O (control) were applied to the membrane for 15-60 min (Step 2 in Figure 5B) and then exposed to Vpp for additional 2 hours (Step 3 in Figure 5B). Finally, 50 μl media were added to release the Vpp on the skin-like gelatin membranes (Step 4 in Figure 5B), and the Vpp activity was evaluated.

The results showed that the coffee leaf extract was coated on the surface of skin-like gelatin membranes within 15-60 minutes, and the effectiveness of wild-type pseudovirus SARS-CoV-2 entry into cells was reduced to under 10%, much stronger suppressive activity than that of 75% Ethanol **(Figure 5C).** These results suggested that coffee leaf extract may have the potential as a hand sanitizer or spray-like disinfectant to prevent SARS-CoV-2 infection.

### Characterization of main compounds in the coffee leaf extracts potentially harboring inhibitory activity against SARS-CoV2

**Figure 6:**
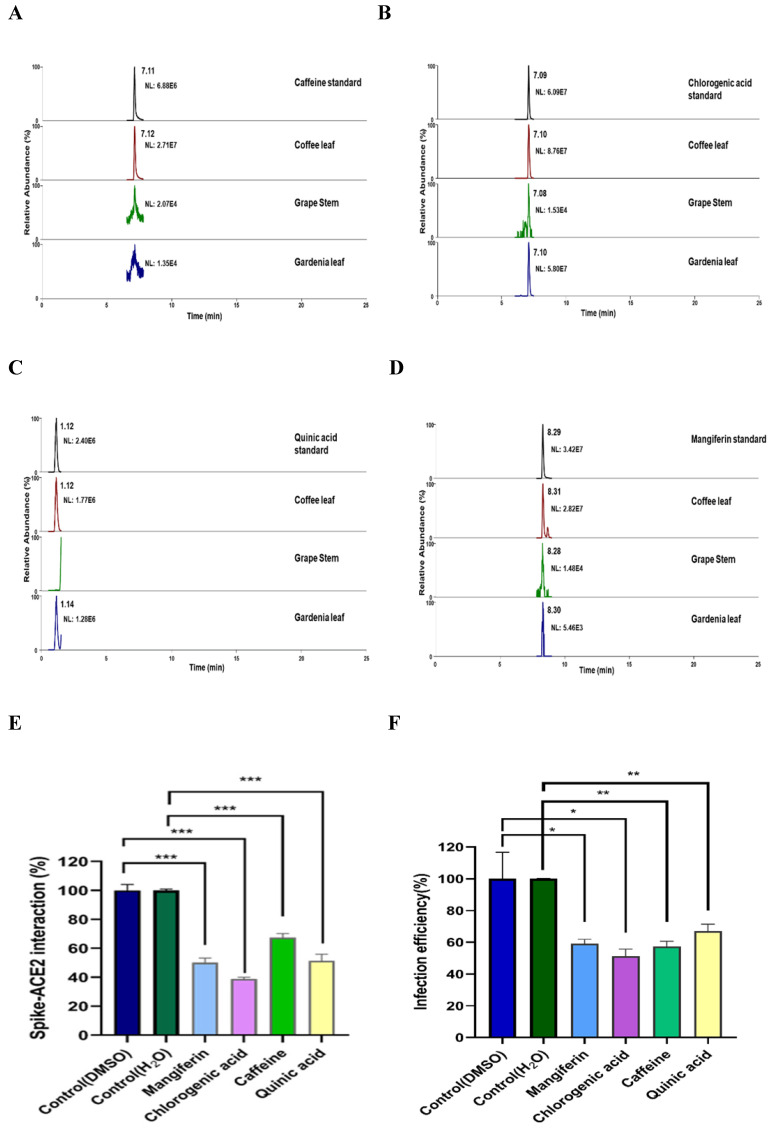
Identification of major compounds of the coffee leaf extract exerts inhibitory activity against interactions of spike-ACE2. Mass ion chromatograms of (A) caffeine (m/z 195.1 → 138.06596), (B) chlorogenic acid (m/z 353.1 → 191.05478), (C) quinic acid (m/z 195.1 → 138.06596), and (D) mangiferin (m/z 421.1 → 301.034836). (E) Mangiferin and CGA can block inhibiting Spike-ACE2 activity measured by the Spike-ACE2 TR-FRET experiment, but caffeine and quinic acid had a weak effect on inhibiting Spike-ACE2 activity. (F) Mangiferin and CGA, caffeine and quinic acid presented inhibition of wild-type SARS-CoV-2 Vpp on 293T-ACE2 cells. Data were presented as mean ± SEM executed in triplicate. Statistical significance was measured by Student t-test, * P < 0.05, ** *P* < 0.01, *** P < 0.001.

To characterize what major compounds in the extracts of these natural plants might harbor the activity to inhibit SARS-CoV-2 infection, we developed the fingerprint of the coffee leaf (*Coffea Arabica*), grape stem (*Vitis vinifera*), and gardenia leaf (*Gardenia jasminoides*) extracts using UHPLC and analyzed by high-resolution mass spectrometer **(Figure 6A-D)**. Consistent with the literature 39-41, four main compounds, including caffeine, CGA, quinic acid, and mangiferin, were identified in the coffee leaf extract **(Table 1)**. Next, we investigated which of the main components of the coffee leaf may prevent SARS-CoV-2 entry. We utilized the TR-FRET assay to inhibit the effectiveness of Spike-ACE2. Due to the nature of the compounds, mangiferin and CGA were soluble in DMSO, and caffeine and quinic acid were soluble in water. The results demonstrated that treatment with four main compounds could inhibit the activity of Spike-ACE2 **(Figure 6E)**. A similar inhibitory effect was also observed by wild-type Vpp assay on 293T-ACE2 cells **(Figure 6F)**. Together, the results indicated that each of the four major components from coffee leaf harbored certain activity to inhibit both Spike-ACE2 interaction and Vpp infectivity and suggest that they may together make the agricultural by-product, coffee extract useful to develop into hand sanitizer or spray-like disinfectants to prevent from SARS-CoV-2 infection.

## Discussion

There are many research articles about the antiviral, antibacterial, and insect repellent activities of natural plants 39, 42. We wanted to find some safe and effective natural plants that will prevent humans from the threat of SARS-CoV-2. It was known that the early step of SARS-CoV-2 infection includes Spike protruding from the virus envelope, providing specificity to interact with host cell receptors (such as ACE2) and host factors (as TMPRSS2). Inhibition of ACE2 or TMPRSS2 receptors can be potential targets for SARS-CoV-2 prevention 43, 44. In this research, we initially obtained three natural plant extracts, grape stem (*Vitis vinifera*), gardenia leaf (*Gardenia jasminoides*), and coffee leaf (*Coffea Arabica*), that can hinder the interaction of Spike-ACE2. Coffee leaf extract can impede Spike-ACE2 protein binding and disturb the activity of the human serine protease TMPRSS2. Furthermore, we validated that the coffee leaf extracts more effectively inhibited wild-type SARS-CoV-2 infecting the host cells and blocking mutant strains' entry through Vpp on 293T-ACE2 cells and Calu-3 cells. It is implied that coffee leaf extract has the potential to inhibit the entry of SARS-CoV-2. In the future, animal models could be executed for in vivo studies to assess the efficacy of coffee leaf extract as an anti-SARS-CoV-2 drug.

Harbourt *et al.* 45 verified that under laboratory conditions, SARS-CoV-2 keeps stable on the skin at 4 °C for 14 days, at 22 °C for at least 96 hours, and at 37 °C for at least 8 hours. Hirose *et al.* 46 additionally reported that the stability of SARS-CoV-2 for 9 hours on human skin may increase the risk of contact transmission, accelerating the pandemic. In the skin-like gelatin membranes experiment, we found that the pseudovirus can be detected even after 6 hours of exposure at room temperature. And also, within 2 hours of exposure, the infected effectiveness did not significantly decline. Ethanol can inactivate SARS-CoV-2 in human skin mucus within 15 seconds. Proper hand hygiene and ethanol disinfectants can lead to rapid inactivation of the virus and may reduce the high risk of contact with the infection 46. However, in 2020, the American Association of Poison Control Center reported that even small amounts of ethanol can lead to confusion, vomiting, breathing stops, and death in children (National Poison Data System, American Association of Poison Control Centers). Moreover, frequent hand sanitizer increases the chance of antimicrobial resistance and other viral diseases 47, 48. There are related studies using compounds or natural plant extracts for topical or skin testing and developing as a barrier to prevent the contact or droplet transmission of SARS-CoV-2. For instance, an insect repellent that contains citric acid is sprayed on the skin, Mosi-guard Natural spray. The authors had identified that Mosi-guard Natural spray SARS-CoV-2 may be against SARS- CoV-2 and has a potential for local prevention 49. Likewise, there is a study showing that HIDROX® cream, water-based olive pulp extracts rich in hydroxytyrosol, applied on the film presents a time- and concentration-dependent anti-SARS-CoV-2 efficacy, which can be used to improve SARS-CoV-2 control measures 50. Here, we used skin-like gelatin membranes to simulate the skin test, and the results show that 100 μg/ml coffee leaf extract can effectively block the SARS-CoV-2 infection within 60 minutes. Even under semi-dry conditions, the effect of impeding the entry of SARS-CoV-2 is much better than the 75% (w/w) Ethanol group. Our research revealed that high coffee leaf extract concentrations might help control SARS-CoV-2, and it is worthy of further exploration for product development.

In many handled coffee leaf extracts, caffeine, CGA, mangiferin, trigonelline, 3-caffeoylquinic acid, 5-caffeoylquinic acid, rutin, 3,4-dicaffeoylquinic acid, and 3,5-dicaffeoylquinic acid were detected 29. Current research revealed that caffeine and CGA are associated with antivirus. For example, caffeine can delay liver fibrosis, hinder the hepatitis C virus (HCV) replication cycle, and improve liver cell function 51. Furthermore, the simulation results indicated that caffeine presented a good binding affinity with SARS-CoV-2 3-chymotrypsin-like protease (3CLpro) 52. CGA is considered the main component of the antioxidant activity of coffee leaves, which can eliminate free radicals and metals and adjust antioxidant enzymes 31, 53. According to reports, 1H-NMR and LC-MS metabolomics analysis revealed that CGA has anti-HSV activity 54. And, the virtual calculation results of molecular docking showed that CGA binds stably to Gln325 and Gln42/Asp38 in ACE2, respectively, and blocks the binding of S-protein to ACE2 55. Compared with the previous report 56, our LC-HRMS analysis of coffee leaf (*Coffea Arabica*) had a similar pattern. Further, we found four major compounds in the coffee leaf (*Coffea Arabica*), such as caffeine, CGA, quinic acid, and mangiferin. In the Spike-ACE2 TR-FRET experiment, CGA seemed to slightly better inhibit Spike-ACE2 than the other three compounds. It is speculated that the activity of unique compounds in the coffee leaf has the potential to block the entry of SARS-CoV-2.

## Conclusions

In conclusion, our research verified that coffee leaf extract potently inhibits Vpp infection through an ACE2-dependent mechanism. It could be a valuable supplement to the daily diet and may also be a promising alternative source for hand sanitizer or spray-like disinfectants to prevent infection of SARS-CoV-2.

## Supplementary Material

Supplementary figure and table.

## Figures and Tables

**Table 1:** LC-HRMS analysis in coffee leaf, grape stem, and gardenia leaf

Sample	Caffeine (mg/g)	CGA (mg/g)	Quinic acid (mg/g)	Mangiferin (mg/g)
Coffee leaf	24.3	7.8	3.5	9.1
Grape stem	N.D.^a^	N.D.	N.D.	N.D.
Gardenia leaf	N.D.	5.5	2.4	N.D.

^a^ N.D.: Not detected LC-HRMS analysis on the content of caffeine, CGA, quinic acid, and mangiferin in coffee leaf (*Coffea Arabica*), grape stem (*Vitis vinifera*), and gardenia leaf (*Gardenia jasminoides*).

## Abbreviations

ACE2: angiotensin-converting enzyme 2
CGA: chlorogenic acid
COVID-19: Coronavirus Disease 2019
FRET: fluorescence resonance energy transfer
SARS-CoV-2: Severe Acute Respiratory Syndrome Coronavirus Type 2
TMPRSS2: transmembrane protease serine 2
VOC: variants of concern
Vpp: SARS-CoV-2 pseudo-particles
