# Supplementary material for: Prospects of Coffee Leaf against SARS-CoV-2 Infection
Source: Int J Biol Sci. 2022 Jul 11;18(12):4677–89. doi: 10.7150/ijbs.76058 (PMC9305275; doi:10.7150/ijbs.76058)
Supplement: Supplementary file 1 — Supplementary figure and table. [file ijbsv18p4677s1.pdf]

Prospects of Coffee Leaf Against SARS-CoV-2 Infection

Chen-Shiou Wu et al.

Supplementary Table 1

The list of our current natural plant extracts which were used to screen by ELISA assay.

ID	Corresponding extracts	ID	Corresponding extracts	ID	Corresponding extracts
#1	***Vitis vinifera*** **(Kyoho) skin, 20% EtOH extract**	#28	***Tetrastigma formosanum*** **leaf, 20% EtOH extract**	#55	***Pueraria lobata,*** **water extract**
#2	***Vitis vinifera*** **(Kyoho) skin, 50% EtOH extract**	#29	***Tetrastigma formosanum*** **leaf, 50% EtOH extract**	#56	***Cinnamomum kanehirae*** **leaf, water extract**
#3	***Vitis vinifera*** **(Kyoho) stem, 20% EtOH extract**	#30	***Litchi chinensis*** **shell, 20% EtOH extract**	#57	***Terminalia catappa,*** **water extract**
#4	***Vitis vinifera*** **(Kyoho) stem, 50% EtOH extract**	#31	***Litchi chinensis*** **shell, 50% EtOH extract**	#58	***Terminalia mantaly,*** **seed water extract**
#5	***Vitis vinifera*** **(Kyoho) leaf, 20% EtOH extract**	#32	***Litchi chinensis*** **seed, 20% EtOH extract**	#59	***Terminalia mantaly,*** **leaf water extract**
#6	***Vitis vinifera*** **(Kyoho) leaf, 50% EtOH extract**	#33	***Litchi chinensis*** **seed, 50% EtOH extract**	#60	***Flemingia macrophylla,*** **water extract**
#7	***Vitis vinifera*** **(Honey Red) skin, 20% EtOH extract**	#34	***Piper betle*** **leaf, 20% EtOH extract**	#61	***Clerodendrum inerme*** **(L.) Gaertn., water extract**
#8	***Vitis vinifera*** **(Honey Red) skin, 50% EtOH**	#35	***Piper betle*** **leaf, 50% EtOH extract**	#62	***Osmanthus fragrans*** **leaf, water extract**

	extract				
#9	*Vitis vinifera* (Honey Red) stem, 20% EtOH extract	#36	*Ampelopsis japonica,* 20 % EtOH extract	#63	*Psychotria rubra,* water extract
#10	*Vitis vinifera* (Honey Red) stem, 50% EtOH extract	#37	*Ampelopsis japonica,* 50 % EtOH extract	#64	*Rosa rugosa* thunb., methanol extract
#11	*Vitis vinifera* (Honey Red) leaf, 20% EtOH extract	#38	*Cestrum nocturnum,* 20 % EtOH extract	#65	*Rosa rugosa,* methanol extract
#12	*Vitis vinifera* (Honey Red) leaf, 50% EtOH extract	#39	*Cestrum nocturnum,* 50 % EtOH extract	#66	*Piper betle* leaf, methanol extract
#13	*Vitis vinifera* (Golden Muscat) stem, 20% EtOH extract	#40	*Antrodia cinnamomea,* 20 % EtOH extract	#67	*Litchi chinensis* shell, methanol extract
#14	*Vitis vinifera* (Golden Muscat) stem, 50% EtOH extract	#41	*Antrodia cinnamomea,* 50 % EtOH extract	#68	*Litchi chinensis* seed, methanol extract
#15	*Vitis vinifera* (Golden Muscat) leaf, 20% EtOH extract	#42	Ixora parviflora Vahl., 20 % EtOH extract	#69	*Coffee Arabica* leaf, methanol extract
#16	*Vitis vinifera* (Golden Muscat) leaf, 50% EtOH extract	#43	*Rhodiola rosea* L., 20 % EtOH extract	#70	*Antrodia cinnamomea,* methanol extract
#17	*Cayratia japonica* (Thunb.) Gagnep stem, 20% EtOH extract	#44	*Pueraria lobata,* 20 % EtOH extract	#71	*Terminalia catappa,* methanol extract
#18	*Cayratia japonica*	#45	*Cinnamomum*	#72	*Terminalia mantaly,* methanol

	**(Thunb.) Gagnep stem, 50% EtOH extract**		***kanehirae* leaf, 20 % EtOH extract**		**extract**
**#19**	***Cayratia japonica* (Thunb.) Gagnep leaf, 20% EtOH extract**	**#46**	***Gardenia jasminoides* leaf, 50 % EtOH extract**	**#73**	***Clerodendrum inerme* (L.) Gaertn., methanol extract**
**#20**	***Cayratia japonica* (Thunb.) Gagnep leaf, 50% EtOH extract**	**#47**	***Barringtonia racemosa,* 50 % EtOH extract**	**#74**	***Ixora parviflora* Vahl., methanol extract**
**#21**	***Vitis amurensis* stem, 20% EtOH extract**	**#48**	**C1 distillers grains, 50 % EtOH extract**	**#75**	***Ixora parviflora* Vahl., fresh leaf methanol extract**
**#22**	***Vitis amurensis* stem, 50% EtOH extract**	**#49**	***Stauntonia keitaoensis* Hayata leaf, 50 % EtOH extract**	**#76**	***Ixora parviflora* Vahl., dried leaf methanol extract**
**#23**	***Coffee Arabica* leaf, 20% EtOH extract**	**#50**	***Vitis vinifera* (Kyoho) stem, water extract**	**#77**	***Psychotria rubra,* methanol extract**
**#24**	***Coffee Arabica* leaf, 30% EtOH extract**	**#51**	***Vitis amurensis* stem, water extract**	**#78**	***Psychotria rubra* stem, methanol extract**
**#25**	***Coffee Arabica* leaf, 50% EtOH extract**	**#52**	***Piper betle,* water extract**	**#79**	***Psychotria rubra* fruit, methanol extract**
**#26**	***Tetrastigma formosanum* stem, 20% EtOH extract**	**#53**	***Litchi chinensis* shell, water extract**		
**#27**	***Tetrastigma formosanum* stem, 50% EtOH extract**	**#54**	***Rhodiola rosea* L., water extract**		

Supplementary Figure 1

A

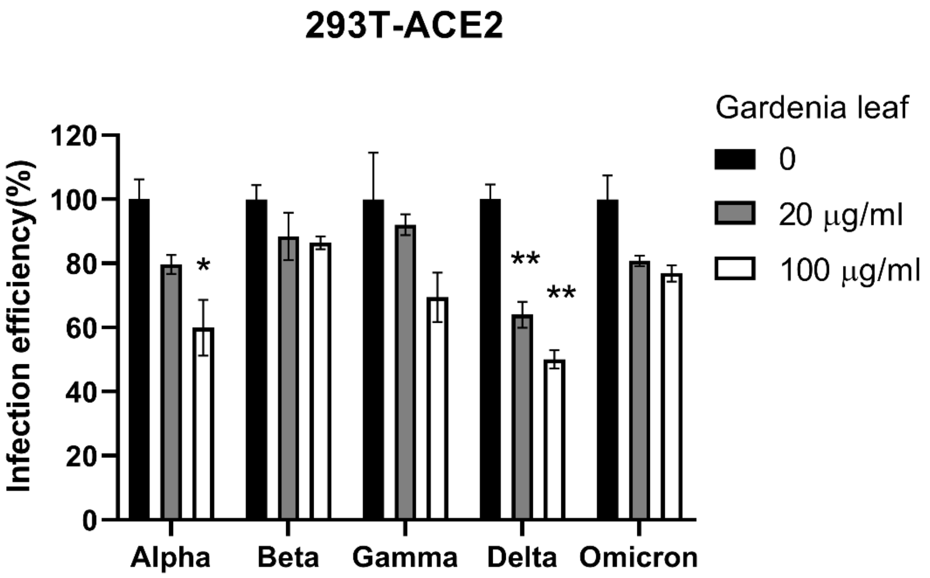

B

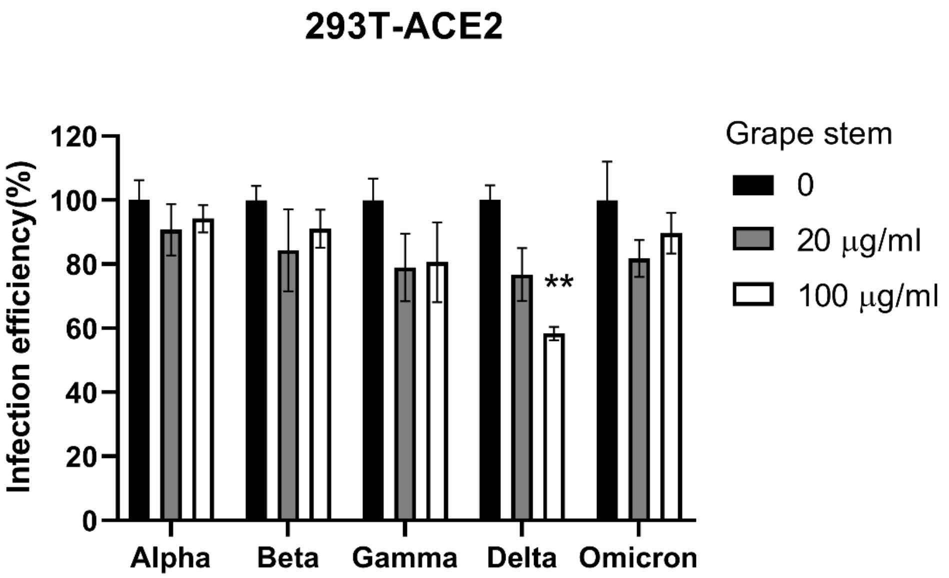

Supplementary Figure 1

(A) The grape stem (Vitis vinifera) extract had the weak effect of inhibiting four VOC SARS-CoV-2 Vpp infections on 293T-ACE2 cells. (B) The gardenia leaf (Gardenia jasminoides) extract had the effect of inhibiting four VOC SARS-CoV-2 Vpp infections on 293T-ACE2 cells. Data were presented as mean ± SEM in triplicate. * $P < 0.05$, ** $P < 0.01$.
